# Pathobiology and Precision Management of Benign and Malignant Bone Tumors: A Narrative Review

**DOI:** 10.7759/cureus.117843

**Published:** 2026-10-13

**Authors:** Abdul Rehman Zia Zaidi, Sara M Samhan, Layan A. Alhammad, Abdullah M. Aldakhil, Abdulaziz M. Al-Mutawa, Mohammed A. Al-Shakhs, Faisal O. Almohaily, Hani A. Alhabdan, Maryah M. Badahdah, Abdullah Y. Asiri, Baraa Alghalyini

**Affiliations:** 1 Department of Family & Community Medicine, College of Medicine, Alfaisal University, Riyadh, SAU; 2 School of Kinesiology and Health Sciences, York University, Toronto, CAN; 3 College of Medicine, Alfaisal University, Riyadh, SAU; 4 College of Applied Medical Sciences, Majmaah University, Majmaah, SAU; 5 College of Medicine, Imam Mohammad Ibn Saud Islamic University, Riyadh, SAU; 6 College of Medicine, King Faisal University, Al-Ahsa, SAU; 7 College of Medicine, King Khalid University, Abha, SAU; 8 College of Medical Rehabilitation Sciences, King Abdulaziz University, Jeddah, SAU

**Keywords:** 3d-printed implants, bone metastases, bone tumors, chondrosarcoma, ewing sarcoma, immunotherapy, limb-salvage surgery, narrative review, osteosarcoma, targeted therapy

## Abstract

Bone tumors range from incidental benign lesions and locally aggressive intermediate tumors to primary sarcomas, and metastatic carcinoma is the most common malignant lesion of adult bone. This narrative review focuses on primary bone tumors, examining their classification, molecular pathobiology, diagnosis, staging, and treatment, and it addresses metastatic bone disease briefly from an orthopedic-oncology perspective. We synthesized literature identified through PubMed, Scopus, Web of Science, and the Cochrane Library, supplemented by targeted verification and updating of cited sources during revision in 2026. Classifications, clinical practice guidelines, clinical trials, cohort studies, and reviews informed the synthesis. Management depends on histological subtype, anatomical site, disease extent, symptoms, and the feasibility of preserving function. Suspected primary bone sarcomas should be imaged locally, staged with chest computed tomography, and referred to a sarcoma center before biopsy. Observation or intralesional treatment is appropriate for many benign lesions, whereas selected locally aggressive tumors require more extensive intervention. Osteosarcoma and Ewing sarcoma generally require systemic chemotherapy and local treatment. Surgery remains central to conventional chondrosarcoma management, and systemic treatment options differ across its subtypes. Molecular findings support, but do not replace, integrated radiological and histopathological diagnosis. Checkpoint inhibitors have shown limited activity in unselected bone sarcomas, and multikinase inhibitors have modest activity in relapsed osteosarcoma. Patient-specific implants, radiomics, liquid biopsy, and cellular therapies are promising, but each requires prospective evidence of improved patient outcomes before broad adoption. Accurate biopsy planning, specialist pathology review, and coordinated sarcoma care remain essential.

## Introduction and background

Orthopedic oncology occupies a distinct position at the junction of surgical oncology, musculoskeletal medicine, and rehabilitation science. The discipline addresses the full clinical arc of bone tumors, from incidental benign findings to metastatic disease requiring palliative stabilization [1,2]. Primary bone malignancies are rare, accounting for fewer than 0.2% of all new cancer diagnoses annually [3,4]. Their clinical impact, however, outweighs their frequency. These tumors disproportionately affect adolescents and young adults during years of peak physical and social development, and they pose surgical challenges that few other malignancies match [5].

The introduction of effective combination chemotherapy alongside surgery substantially improved outcomes in localized osteosarcoma [6,7]. Developments in imaging, resection planning, and reconstruction have expanded limb-sparing treatment. Endoprostheses can preserve function after tumor resection, although infection, mechanical failure, and revision surgery remain important long-term concerns [8].

Yet progress has been uneven. Five-year survival for localized osteosarcoma has remained near 60 to 70% for roughly three decades, a plateau that has resisted successive clinical trials [9]. Ewing sarcoma cure rates for localized disease approach 70%, but metastatic Ewing sarcoma still carries a five-year survival below 30% [10]. Conventional chondrosarcoma has limited sensitivity to cytotoxic chemotherapy, and surgery remains the principal treatment for resectable disease [11]. Metastatic bone disease from carcinomas of the breast, prostate, lung, kidney, and thyroid vastly outnumbers primary bone tumors [12].

Patient-specific 3D-printed titanium implants now allow anatomically precise reconstruction after complex tumor resections [13]. Molecular findings incorporated in the 2020 WHO classification now support diagnoses such as H3-3A (H3F3A)-mutant giant cell tumor of bone and Ewing sarcoma defined by a FET::ETS gene fusion, most often EWSR1::FLI1 [14-16]. Immunotherapy is being explored through chimeric antigen receptor (CAR) T-cell platforms and combination regimens [17,18]. Artificial intelligence (AI) and radiomics are being investigated for tumor characterization and treatment-response prediction [19,20]. Liquid biopsy approaches, including circulating tumor DNA (ctDNA) and cell-free RNA, are also under investigation for sarcoma monitoring [21,22].

Bone lesions fall into four broad groups with different clinical implications. Benign lesions, such as osteochondroma, enchondroma, and non-ossifying fibroma, are often found incidentally and frequently need only observation. Intermediate, locally aggressive tumors, typified by giant cell tumor of bone, rarely metastasize but can destroy bone and recur after treatment. Primary malignant bone tumors, chiefly osteosarcoma, Ewing sarcoma, and chondrosarcoma, require specialist multimodal care. Metastatic carcinoma is the most common malignant lesion of adult bone and is treated according to the primary cancer, with orthopedic input focused on diagnosis, fracture prevention, stabilization, and palliation [12,14].

This review focuses on primary bone tumors, benign, intermediate, and malignant, and considers metastatic bone disease only where it raises orthopedic-oncology decisions. It addresses four clinical questions: how current classification and molecular pathology inform diagnosis; which diagnostic sequence, from imaging through referral, biopsy, and staging, avoids compromising definitive treatment; what evidence supports current surgical, systemic, and radiation treatment for the main primary bone tumors; and which emerging diagnostic and therapeutic approaches are ready for clinical use and which remain investigational. The review was structured in accordance with the Scale for the Assessment of Narrative Review Articles (SANRA) checklist [23].

## Review

Methods

We searched PubMed, Scopus, Web of Science, and the Cochrane Library; the searches were completed on 10 and 12 September 2026. They covered publications from January 2000 through March 2025, with topic-specific updates through December 2025. Searches combined terms for bone tumors, osteosarcoma, Ewing sarcoma, chondrosarcoma, bone metastases, orthopedic oncology, limb salvage, 3D-printed implants, immunotherapy, and targeted therapy. Database-specific search strings were not archived, so the search cannot be reproduced exactly; the term sets above are reported instead. Earlier foundational publications were retained where they defined classification, staging, or treatment development.

Sources considered included clinical practice guidelines, classification documents, randomized and nonrandomized clinical trials, systematic reviews, large cohort or registry studies, and primary studies directly relevant to the diagnosis or management of bone tumors. Preclinical studies were cited only where they were the main evidence for an emerging approach and were identified as preclinical in the text. Screening was shared across the author team: pairs of authors screened titles and abstracts for the sections assigned to them, reviewed the full texts of potentially relevant sources, and resolved disagreements by discussion.

We did not perform a formal risk-of-bias assessment. When sources addressed the same question, we gave priority to clinical practice guidelines and randomized trials, then to prospective nonrandomized trials and systematic reviews, and then to large observational studies. Quantitative pooling was not undertaken because the studies differed in tumor type, intervention, design, and outcome. Evidence was compared qualitatively, and effect estimates and 95% confidence intervals (CIs) are reported as published.

During revision, we verified each cited trial result against its primary report and added current clinical practice guidelines and the primary trials of agents discussed in the text, including reports published in 2026. This verification was targeted and was not a further systematic search. This was a narrative synthesis rather than a systematic review, and it was not designed to be reproducible. The selection of sources is therefore open to selection bias, and the review does not claim completeness. The SANRA informed the presentation of the review [23]. No new participant data were collected.

Classification of bone tumors

The WHO Classification of Tumours of Soft Tissue and Bone (fifth edition, 2020) provides the most widely adopted framework [14]. It groups bone neoplasms into benign, intermediate (locally aggressive or rarely metastasizing), and malignant categories, each subdivided by tissue of origin. Molecular findings support, but do not replace, an integrated diagnosis based on clinical, radiological, and histopathological features [14,16].

Benign and Intermediate Bone Tumors

Most benign bone tumors grow slowly and remain well-circumscribed [24]. Osteochondroma is the most common benign bone tumor, accounting for approximately 20% to 50% of benign osseous lesions in reported series [14,25]. Hereditary multiple osteochondromas are associated with EXT1 or EXT2 alterations; reported malignant transformation risk is approximately 3% to 5% [14,25].

Enchondroma commonly involves the small tubular bones of the hands and feet. Multiple enchondromas in Ollier disease or Maffucci syndrome confer an increased risk of chondrosarcoma [14]. IDH1 and IDH2 mutations occur early in the development of central cartilaginous tumors and can generate the oncometabolite 2-hydroxyglutarate [26]. Their therapeutic relevance is supported by studies of IDH1 inhibition in selected advanced chondrosarcomas [27].

Non-ossifying fibroma is a benign developmental lesion with a characteristic radiographic evolution [28]. Giant cell tumor of bone (GCTB) belongs to the intermediate, locally aggressive category and is characterized by H3-3A (H3F3A) mutations [14,15]. Its neoplastic stromal cells express RANK ligand, promoting recruitment of osteoclast-like giant cells and osteolysis; this provides the biological rationale for denosumab treatment in selected patients [29].

Malignant Bone Tumors

Osteosarcoma is the most common primary malignant bone tumor, with an annual incidence of roughly three to four cases per million [3,30]. Its molecular profile is defined by genomic instability rather than a single driver mutation [31,32]. Ewing sarcoma, the second most frequent bone malignancy in youth, is defined by a fusion between a FET-family gene, usually EWSR1, and an ETS-family transcription factor gene; EWSR1::FLI1 occurs in about 85% of cases and EWSR1::ERG in about 10% [10,16,33]. Five-year survival for localized disease is near 70%; for metastatic disease, it is below 30% [10,34].

Conventional chondrosarcoma usually affects adults and is relatively resistant to cytotoxic chemotherapy and conventional radiotherapy. Surgery is the principal treatment for resectable disease; unresectable disease has a poor prognosis [11,35]. Terminology depends on site: low-grade central cartilaginous tumors of the appendicular skeleton are termed atypical cartilaginous tumor, whereas histologically similar tumors of the axial skeleton, including the pelvis, scapula, and skull base, are termed chondrosarcoma grade 1 [14,16]. IDH1 or IDH2 mutations occur in a substantial proportion of central cartilaginous tumors, while COL2A1 alterations provide additional evidence of disrupted cartilage biology [26,36]. An international consensus found no role for routine chemotherapy in chondrosarcoma, but treatment decisions must distinguish conventional disease from rarer subtypes and account for resectability [37].

Epidemiology and risk factors

Benign bone tumors outnumber malignant ones by a wide margin and arise most often during rapid skeletal growth [14,38]. Annual incidence of primary malignant bone tumors in Western populations is approximately 0.8 to 1.0 per 100,000 person-years [4]. The American Cancer Society estimated 3970 new cases and 2050 deaths from bone and joint cancers in the United States in 2024 [3].

Osteosarcoma shows an adolescent incidence peak and a later peak in older adults [30]. The older population includes tumors associated with Paget disease or prior irradiation [39]. Men are affected more often than women [40]. Ewing sarcoma is more frequent in populations of European ancestry than in populations of East Asian or African ancestry [10,33]. Predisposition syndromes include Li-Fraumeni syndrome, hereditary retinoblastoma, and disorders of DNA repair [39,41,42]. Multiple osteochondromas and enchondromatosis syndromes also carry risks of malignant transformation [14,26].

Clinical presentation and diagnostic evaluation

Symptomatology

Pain, swelling, and functional impairment constitute the classic triad, but their quality and tempo differ between benign and malignant lesions [43]. Pain from malignant tumors is typically progressive, present at rest, worse at night, and poorly responsive to standard analgesics [44].

Imaging, Referral, and Staging

Plain radiography is the first investigation and assesses tumor location, matrix mineralization, periosteal reaction, cortical destruction, and lesion margins [45]. When radiographs cannot exclude malignancy, magnetic resonance imaging (MRI) of the whole involved compartment, including the adjacent joints, defines intraosseous and soft-tissue extent and should be completed before biopsy [16,46]. A lesion that remains suspicious for a primary bone sarcoma should then be referred to a sarcoma reference center, where the initial work-up, including biopsy, takes place [16]. Staging for distant disease should include chest computed tomography (CT) to detect pulmonary metastases, together with bone scintigraphy, whole-body MRI, or fluorodeoxyglucose positron emission tomography/CT (FDG PET/CT) as indicated [16,46,47]. FDG PET/CT can also contribute to treatment-response assessment in selected bone sarcomas [48,49].

Biopsy and Histopathology

Biopsy should be performed by the surgical team that will undertake the definitive resection, or by an interventional radiologist after discussion with that team, because a poorly placed biopsy tract can compromise later resection and limb preservation [16,50,51]. The tract should be placed so that it can be excised at definitive surgery, and sampling should provide enough viable tissue for histological and molecular testing [52]. Core needle biopsy is often sufficient, but diagnostic performance depends on sampling and the outcome assessed. In one study, a single needle biopsy classified benign versus malignant lesions with 92.4% accuracy, whereas exact pathological diagnosis was less accurate [53]. Histology should therefore be interpreted alongside imaging and appropriate molecular testing. The WHO framework incorporates H3-3A (H3F3A) mutations in GCTB, FET::ETS fusions in Ewing sarcoma, and IDH1 or IDH2 mutations in central cartilaginous tumors, but these findings support rather than replace integrated radiological and histopathological diagnosis [14,15].

Staging and prognostication

The Enneking system, adopted by the Musculoskeletal Tumor Society, describes histological grade, anatomical compartment, and metastatic status [54]. The American Joint Committee on Cancer (AJCC) eighth edition, which remains the current edition for bone tumors, and the corresponding Union for International Cancer Control TNM classification describe tumor size or extent, nodal and distant metastases, and grade, with separate T categories for the spine and pelvis and a distinction between pulmonary and other distant metastases [55,56]. The Enneking classification also distinguishes latent, active, and aggressive benign lesions and stages malignant tumors according to grade, local extent, and metastases; it remains widely used for surgical planning [54,57,58]. Staging supports treatment planning but does not replace histology-specific assessment. Prognosis also depends on age, tumor size, anatomical site, chemotherapy response, and surgical margins [59-61]. In osteosarcoma, extensive necrosis after preoperative chemotherapy is associated with better outcomes, although EURAMOS-1 did not demonstrate benefit from the tested chemotherapy intensification in poor responders [9,60].

Treatment strategies

Current treatment recommendations for bone sarcomas are summarized in the ESMO-EURACAN-GENTURIS-ERN PaedCan and NCCN clinical practice guidelines [16,47]. The sections below summarize the evidence underlying the main treatment choices.

Surgical Management

Surgery remains the primary modality for local control. Limb-sparing surgery is an established option when adequate oncologic margins and a functional reconstruction are achievable; comparisons with amputation must account for differences in patient and tumor characteristics [8,61,62]. Reconstruction options include endoprostheses, structural allografts, and patient-specific 3D-printed titanium implants [13,63-65]. A small retrospective comparison of long-term survivors found better quality-of-life measures after rotationplasty than after amputation; selection and survivorship differences limit causal interpretation [66]. Management of benign lesions depends on diagnosis, symptoms, fracture risk, and biological behavior, with options ranging from observation to intralesional treatment or resection [24,58].

Chemotherapy

For osteosarcoma, the standard methotrexate, doxorubicin, and cisplatin (MAP) regimen has not been improved upon. The EURAMOS-1 trial randomized 618 patients with a poor histological response and found no event-free survival benefit from adding ifosfamide and etoposide (hazard ratio (HR) 0.98, 95% CI 0.78 to 1.23), with greater toxicity [9]. Standard frontline therapy for pediatric bone sarcomas has not materially changed in decades [67]. For Ewing sarcoma, vincristine, doxorubicin, and cyclophosphamide alternating with ifosfamide and etoposide (VDC/IE) is a standard regimen. For relapsed or refractory Ewing sarcoma, the multi-arm rEECur trial recruited 451 patients; in its phase III comparison of topotecan-cyclophosphamide with high-dose ifosfamide, 73 patients were randomized to each arm. Median event-free survival was 3.7 months (95% CI 2.1 to 6.2) with topotecan-cyclophosphamide and 5.7 months (95% CI 3.8 to 7.0) with high-dose ifosfamide, and median overall survival was 10.4 and 16.8 months, respectively; the Bayesian analysis gave a probability of about 95% that ifosfamide was superior for both outcomes [68]. These results are available only as a conference abstract. Conventional chondrosarcoma has limited sensitivity to cytotoxic chemotherapy. Mesenchymal and dedifferentiated subtypes require separate consideration; evidence for systemic treatment differs between them [11,37].

Radiotherapy

Ewing sarcoma is radiosensitive [10]. Carbon-ion radiotherapy has been studied for unresectable sacral chordoma [69]. The role of radiotherapy in metastatic bone disease is discussed separately below.

Bone-Targeted Agents

In GCTB, an initial phase II denosumab study enrolled 37 patients, of whom 35 were evaluable; 30 (86%, 95% CI 70 to 95) met its composite response endpoint of marked giant-cell depletion or absence of radiographic progression [29]. This endpoint should not be equated with an objective radiographic response rate. A subsequent phase II study enrolled 282 patients. Among patients with surgically unsalvageable disease, 163 of 169 (96%) had no disease progression, and among those whose planned surgery would have caused severe morbidity, 74 of 100 (74%) had not undergone surgery at the interim analysis. Osteonecrosis of the jaw (1%) and hypocalcemia (5%) were among the reported adverse events [70].

Immunotherapy and Targeted Therapy

In the SARC028 trial, two of 40 evaluable patients with bone sarcoma (5%) had an objective response to pembrolizumab [71]. Correlative analyses identified TGF-beta signaling, interferon-pathway activation, and low HLA class I expression as potential contributors to checkpoint resistance in Ewing sarcoma [72]. In IMMUNOSARC, 40 patients received nivolumab plus sunitinib, and 38 were evaluable by central radiological review; the six-month progression-free survival rate was 42% (95% CI 27 to 58), the objective response rate was 5%, and one patient died of treatment-related pneumonitis [73].

CAR T-cell approaches are being investigated in sarcomas, including clinical studies targeting HER2 and preclinical work targeting folate receptor-alpha in osteosarcoma [74,75]. Multikinase inhibitors have shown activity in relapsed osteosarcoma [76]. In the placebo-controlled phase II SARC024 trial (42 patients with metastatic osteosarcoma), regorafenib prolonged median progression-free survival from 1.7 to 3.6 months (HR 0.42, 95% CI 0.21 to 0.85), without a significant difference in overall survival [77]. In the osteosarcoma cohort of the REGOBONE trial, 17 of 26 evaluable patients (65%) receiving regorafenib were free of progression at eight weeks, compared with none of 12 receiving placebo [78]. In the single-arm CABONE trial, cabozantinib produced objective responses in 5 of 42 assessable patients with osteosarcoma (12%, 95% CI 4 to 26) and 10 of 39 with Ewing sarcoma (26%, 95% CI 13 to 42); 33% (95% CI 20 to 50) of patients with osteosarcoma were free of progression at six months [79]. In a 262-patient observational cohort, median time to next treatment was 7.6 months (95% CI 5.1 to 12.5) for tyrosine kinase inhibitors used in the first line; this estimate does not represent the entire cohort [80].

In phase I testing of ivosidenib, long-term follow-up reported objective responses in 3 of 13 patients with IDH1-mutant conventional chondrosarcoma (23.1%, 95% CI 5.0 to 53.8), with a median response duration of 53.5 months (range 25.8 to 67.9) [27,81]. The randomized, placebo-controlled phase III CHONQUER trial (NCT06127407) is evaluating ivosidenib in this population [82]. In the INT-0133 trial of 662 patients with osteosarcoma, adding mifamurtide to chemotherapy improved six-year overall survival from 70% to 78% (HR 0.71, 95% CI 0.52 to 0.96) [83].

Multidisciplinary team approach

Treatment coordinated through specialized multidisciplinary sarcoma services has been associated with better survival in observational studies [84,85]. A survey of 192 specialists from 47 countries found that 40% reported that most patients in their countries received treatment at specialized sarcoma centers [86]. Table 1 summarizes the major primary malignant bone tumors, and Table 2 presents selected clinical trials relevant to their management.

**Table 1:** Summary of major primary malignant bone tumors CK, cytokeratin; IDH, isocitrate dehydrogenase; yr, years. Features are representative and vary by subtype and site [10,11,14,16,43].

Feature	Osteosarcoma	Ewing Sarcoma	Chondrosarcoma	Chordoma	Adamantinoma
Peak age	10-25; >60 yr	5-25 yr	40-70 yr	50-70 yr	20-50 yr
Common sites	Distal femur, proximal tibia	Pelvis, femur, ribs	Pelvis, proximal femur	Sacrum, skull base	Tibial diaphysis
Key molecular feature	Complex genome; TP53/RB1 alterations	FET::ETS fusion (EWSR1::FLI1 in about 85%)	IDH1/IDH2 mutations (central tumors)	Brachyury expression	CK (epithelial markers)
Chemosensitivity	Moderate	High	Low (conventional subtype)	Limited	Limited

**Table 2:** Selected clinical trials in the management of primary bone tumors * The rEECur entry summarizes the cited ASCO 2022 conference abstract; a full peer-reviewed report was not available. CCR, Clinical Cancer Research; CI, confidence interval; DOR, duration of response; EFS, event-free survival; GCTB, giant cell tumor of bone; HR, hazard ratio; IFOS, high-dose ifosfamide; JCO, Journal of Clinical Oncology; MAP, methotrexate, doxorubicin, and cisplatin; MAPIE, MAP plus ifosfamide and etoposide; MTP, muramyl tripeptide (mifamurtide); ORR, objective response rate; OS, overall survival; PFS, progression-free survival; RCT, randomized controlled trial; TC, topotecan-cyclophosphamide. Enrolled denotes patients who entered the trial; analyzed or evaluable denotes patients with sufficient data for the specified analysis. For IMMUNOSARC, this required imaging available for central radiological review. For the initial denosumab study, this required sufficient histological or radiological data to assess the composite response endpoint. Conventional refers to the conventional histological subtype of chondrosarcoma.

Trial	Authors	Year	Journal	Patients enrolled; analyzed	Key finding	Design
EURAMOS-1 [9]	Marina et al.	2016	Lancet Oncol.	618 poor responders randomized	MAPIE did not improve EFS vs. MAP (HR 0.98, 95% CI 0.78 to 1.23); 3-year EFS 53% vs. 55%; more toxicity with MAPIE	Phase 3 RCT
rEECur, TC vs. IFOS comparison [68]	McCabe et al.	2022*	JCO (abstract)	451 recruited to the multi-arm trial; 73 per arm in the TC vs. IFOS comparison	Median EFS 5.7 months (95% CI 3.8 to 7.0) with IFOS vs. 3.7 months (95% CI 2.1 to 6.2) with TC; median OS 16.8 vs. 10.4 months; posterior probability about 95% favoring IFOS	Phase 3 RCT (multi-arm, Bayesian)
INT-0133 [83]	Meyers et al.	2008	JCO	662 randomized	Adding MTP improved 6-year OS (78% vs. 70%; HR 0.71, 95% CI 0.52 to 0.96)	Phase 3 RCT
SARC028, bone cohort [71]	Tawbi et al.	2017	Lancet Oncol.	86 enrolled in trial; 42 bone sarcoma treated; 40 evaluable	ORR 5% (2 of 40) with pembrolizumab; CI not reported for the bone cohort	Phase 2, single-arm
IMMUNOSARC [73]	Palmerini et al.	2025	Cancer	40 enrolled; 38 evaluable by central review	6-month PFS 42% (95% CI 27 to 58); ORR 5% (2 of 38); one fatal pneumonitis	Phase 2, single-arm
SARC024 [77]	Davis et al.	2019	JCO	42 randomized	Regorafenib vs. placebo: median PFS 3.6 vs. 1.7 months (HR 0.42, 95% CI 0.21 to 0.85); no significant OS difference	Phase 2 RCT
REGOBONE, osteosarcoma cohort [78]	Duffaud et al.	2019	Lancet Oncol.	43 enrolled; 38 evaluable (26 regorafenib, 12 placebo)	Free of progression at 8 weeks: 17 of 26 (65%) vs. 0 of 12	Phase 2 RCT, non-comparative
CABONE [79]	Italiano et al.	2020	Lancet Oncol.	90 enrolled (45 osteosarcoma, 45 Ewing); 42 and 39 assessable	Osteosarcoma: ORR 12% (95% CI 4 to 26), 6-month non-progression 33% (95% CI 20 to 50); Ewing sarcoma: ORR 26% (95% CI 13 to 42)	Phase 2, single-arm
Ivosidenib phase 1 [27,81]	Tap et al.	2020; 2025	JCO; CCR	21 with chondrosarcoma; 13 with conventional histology	Conventional chondrosarcoma: ORR 23.1% (95% CI 5.0 to 53.8); median DOR 53.5 months (range 25.8 to 67.9)	Phase 1
Denosumab in GCTB [29,70]	Thomas et al.; Chawla et al.	2010; 2013	Lancet Oncol.	2010: 37 enrolled, 35 evaluable; 2013: 282 enrolled	2010: 30 of 35 (86%, 95% CI 70 to 95) met the composite endpoint; 2013: no progression in 163 of 169 (96%) with unsalvageable disease; 74 of 100 (74%) with salvageable disease avoided surgery	Phase 2

Metastatic bone disease: orthopedic considerations

Metastatic carcinoma outnumbers primary bone tumors by roughly 25:1 in adults, and the most frequent primary sources are breast, prostate, lung, kidney, and thyroid carcinomas [12,87]. Its distribution follows Paget's seed-and-soil hypothesis, and disrupted bone remodeling produces lytic, sclerotic, or mixed lesions and skeletal-related events [12,88-90]. For the orthopedic oncologist, four questions dominate: whether the lesion is metastatic at all, whether it is likely to fracture, whether it needs stabilization, and how symptoms should be palliated.

A new bone lesion should not be assumed to be metastatic, even in a patient with known cancer. A primary bone sarcoma that is fixed as if it were a metastasis may lose the option of limb-sparing resection, so a lesion that could be a primary sarcoma should follow the referral and biopsy principles described above [16]. For skeletal metastases of unknown origin, a prospective diagnostic strategy of history, examination, laboratory tests, chest radiography, bone scintigraphy, CT of the chest, abdomen, and pelvis, and biopsy identified the primary site in 34 of 40 patients (85%) [91].

Fracture risk in long-bone metastases is commonly estimated with the Mirels score, which combines lesion site, pain, radiographic type, and size [92]. Newer decision tools are being evaluated, and prophylactic stabilization is advised when an impending fracture is identified [93]. Postoperative radiotherapy is recommended after surgery for nonspine bone metastases, and external beam radiotherapy is an established treatment for painful bone metastases [12,94]. Bisphosphonates and denosumab can reduce skeletal-related events [90]. In the phase II SABR-COMET trial, 99 patients with oligometastatic cancers of mixed primary sites were randomized; median overall survival was 50 months with stereotactic ablative radiotherapy and 28 months with standard care (HR 0.47, 95% CI 0.27 to 0.81) [95]. This mixed-cancer population provides indirect evidence only and does not establish benefit in primary bone sarcomas.

Discussion

Chemotherapy and advances in local treatment have improved outcomes in osteosarcoma and Ewing sarcoma, but survival for localized disease has changed little in recent decades, and chemotherapy intensification for poor responders did not help in EURAMOS-1 [9,67]. Further progress is more likely to come from new agents tested in biologically defined populations, in trials designed to show effects on survival rather than response alone.

Relapsed and refractory Ewing sarcoma remains difficult to treat despite sensitivity to chemotherapy [10]. The rEECur program provides the first randomized comparison of salvage regimens, but its results have not yet been published as a full peer-reviewed report [68]. A Euro-Ewing Consortium review of phase I/II trials reported an average disease control rate of 44% and an average response rate of 8% across heterogeneous interventions [96]; such aggregate figures cannot establish the relative efficacy of individual agents. International randomized trials are needed to determine which targeted or immune-based strategies improve outcomes.

Chondrosarcoma management depends on subtype, grade, and site. The BOOM consensus identified broad agreement on several management questions but persistent uncertainty over atypical cartilaginous tumors and surgical margins [37]. Ivosidenib is the first molecularly selected option for IDH1-mutant conventional chondrosarcoma, but the supporting evidence comes from a small nonrandomized cohort, and CHONQUER will determine whether durable responses translate into a progression-free survival benefit [82].

Several emerging approaches share the same evidentiary limitation. Patient-specific 3D-printed implants can address complex pelvic and periarticular defects, but comparative evidence is retrospective, and infection, revision, function, and long-term durability remain to be established [13,64,65]. Reconstruction choices, including rotationplasty for selected patients, should incorporate patient preferences as well as anticipated function and revision burden [66]. Radiomics and deep learning are being evaluated for tumor characterization and response prediction [19,20]; in one retrospective study of 867 whole-slide images from three medical centers and the Children's Oncology Group, a computational pipeline distinguished Ewing sarcoma from other non-rhabdomyosarcoma soft-tissue sarcomas with an area under the receiver operating characteristic curve of 0.929 [97]. Liquid biopsy may support disease monitoring, but its clinical role remains under evaluation [21], and the plasma cell-free RNA data cited here come from a 10-patient series of mixed histologies [22]. Single-agent checkpoint blockade has shown limited activity in unselected bone sarcomas, proposed resistance mechanisms are not validated selection biomarkers, and combination therapy has added toxicity without clear evidence of greater benefit [71-73]. Cellular therapies remain at early clinical or preclinical stages [74,75]. For each of these approaches, the next step is prospective evaluation showing improved patient outcomes before broad adoption.

Health equity is an increasingly recognized dimension of bone tumor management. In a retrospective osteosarcoma study, Black race and Hispanic ethnicity were associated with treatment delays exceeding one month in the subgroup younger than 40 years. Longer delays were associated with lower five-year and 10-year disease-specific survival, without establishing causality [98]. Greater county-level social vulnerability was associated with shorter surveillance periods across several bone cancer histologies; shorter survival periods were reported for chondrosarcoma and chordoma [99]. Access to specialized sarcoma centers is uneven across countries [86], and the United States hosts over 60% of all osteosarcoma clinical trials while low- and middle-income countries, which carry 40% of the global disease burden, account for fewer than 2% [100]. Access to timely specialist care and clinical trials therefore remains an important component of efforts to improve outcomes.

This review has several limitations. The narrative approach and the absence of a formal risk-of-bias assessment introduce potential selection bias, the search strings were not archived, and the verification during revision was targeted rather than systematic, so relevant studies may have been missed. The breadth of the topic limits the depth for any single entity. Some cited results, including the rEECur comparison, are available only as conference abstracts. Many emerging therapies discussed, including CAR T-cell approaches, liquid biopsy, and AI-based diagnostics, are supported by early-phase data with limited follow-up, and their clinical impact remains to be determined.

## Conclusions

Bone tumor management requires accurate classification and treatment tailored to histology, location, disease extent, and functional priorities. Local imaging, early referral, biopsy planning, chest CT staging, and pathology review within a specialist multidisciplinary service are central to avoiding preventable diagnostic and surgical problems. Molecular testing and patient-specific reconstruction can refine care, but new diagnostic and therapeutic approaches should be judged by their effects on patient outcomes. The main research priorities are comparative trials for high-risk disease, prospective validation of biomarkers and computational diagnostics, and better access to specialist services. These priorities should guide adoption of emerging technologies while preserving established principles of oncologic surgery and systemic treatment.
